# Evaluation of beneficial effect of a dual-task exercise based on Japanese transitional games in older adults: a pilot study

**DOI:** 10.18632/aging.103908

**Published:** 2020-10-11

**Authors:** Jieun Yoon, Hiroko Isoda, Tomohiro Okura

**Affiliations:** 1 R&D Center for Tailor-Made QOL, University of Tsukuba, Tsukuba, Ibaraki, Japan; 2 Alliance for Research on the Mediterranean and North Africa (ARENA), University of Tsukuba, Tsukuba, Ibaraki, Japan; 3 Faculty of Life and Environmental Sciences, University of Tsukuba, Tsukuba, Ibaraki, Japan; 4 Faculty of Health and Sport Science, University of Tsukuba, Tsukuba, Ibaraki, Japan

**Keywords:** dual-task exercise, rock-paper-scissors (RPS) game, physical function, cognitive function

## Abstract

Not only does Japan has the world’s longest healthy life expectancy, but also the world’s longest average life span. This study investigated the effect of a novel dual-task (DT) exercise called “Synapsology” (SYNAP), developed as a game-like activity to improve older adults’ physical and cognitive functions. Participants (n=24) with a mean age of 70.6 years (65–77 years) were randomly assigned to the SYNAP exercise group (SG, n=15) and the control group (CG, n=9). The SG participated in the DT intervention consisting of 60-minute sessions, twice a week, for 8 weeks. Physical function in timed-up-and-go had significant pre- and post- trial differences (P=0.017) in SG. In addition, cognitive function results in the a 25-hole trail-making peg test (P=0.004) and an oxidative stress marker (P=0.039) had a statistically significant difference within the SG. However, there were no significant differences in the physical and cognitive functions between SG and CG. In the study, older adults who participated in cognitive-motor DT intervention improved significantly with regard to motor ability and cognitive function results. Thus, a game-like DT exercise may help maintain the healthy life of older adults compared to no intervention.

## INTRODUCTION

According to the World Health Organization (WHO) report (2016), the population of Japan has the longest average human life span at 83.7 years [1]. The population of adults aged over 65 reached 34.61 million in 2017, which comprised 27.3% of the total population. Japan has become a “super-aging” society. As the elderly population has grown faster than expected, the burden of social care insurance on the public due to the welfare policy implemented since 2001 has increased. Therefore, the extension of “healthy lifespan” that aim to maintain a healthy life among Japanese is one of the major goals of the Japanese government.

In 2012, the estimated number of adults over 65 years suffering from dementia in Japan reached 4.42 million, which was about 1 in 7 people with dementia (prevalence rate 15.0%) [2]. Moreover, the estimate for people who may suffer from dementia in 2025 is 37%. That is 1 in every 5 people with dementia. Therefore, the risk of cognitive impairment in older adults is a serious problem in Japan.

Since older people with a risk of chronic disease are often prescribed several medications, they also have a greater risk of adverse medication events, and they may experience side effects [3]. Therefore, the ideal treatment for them may be a combination of natural supplements and exercise, instead of medications [4].

Older adults experience a decrease in physical and social activities due to physical, physiological, and cognitive changes [5]. Several observational studies on older adults revealed that greater physical activity reduces the incidence of dementia [6, 7]. Recently, some reports claimed that physical exercise is associated with improved exercise in various cognitive domains, including novel object recognition memory [8], and cognitive flexibility [9]. Furthermore, studies show that interventional exercises not only improve the essential physical elements needed to avoid falling, such as balance, muscle strength, and agility, but also the cognitive function, even when started at the age where one is most prone to dementia [10]. A meta-analytic study on exercise and cognition indicated that an evaluation result is dependent upon cognitive assessment instruments, exercise protocols (including type of exercise, length, and intensity of exercise programs), and subject-selection criteria [11]. In this respect, the authors suggested performing larger and better controlled studies to characterize important individual differences that moderate exercise produces on cognitive and brain health. Using both cognitive and motor assessments would allow the clinician to make better estimates of functional exercise as seen in other neurocognitive disorders, such as Parkinson’s disease and dementia [12].

Dual-task (DT) exercise is said to be followed when specific cognitive and/or motor tasks are performed simultaneously with the goal of improving selected cognitive or physical domains and it is better than performing the individual exercise or cognitive training alone [13, 14]. Because of this, cognitive-motor DT programs are an emerging modality for reaping the largest cognitive health benefits.

Synapsology (SYNAP) program is a novel, game-like, DT exercise program that is both safe and effective for older adults with cognitive dysfunction [15] when compared with the conventional DT study, which consists of simple tasks, such as stepping exercise while sitting, combined with word enumerating exercise [16–18].

**Figure 1:**
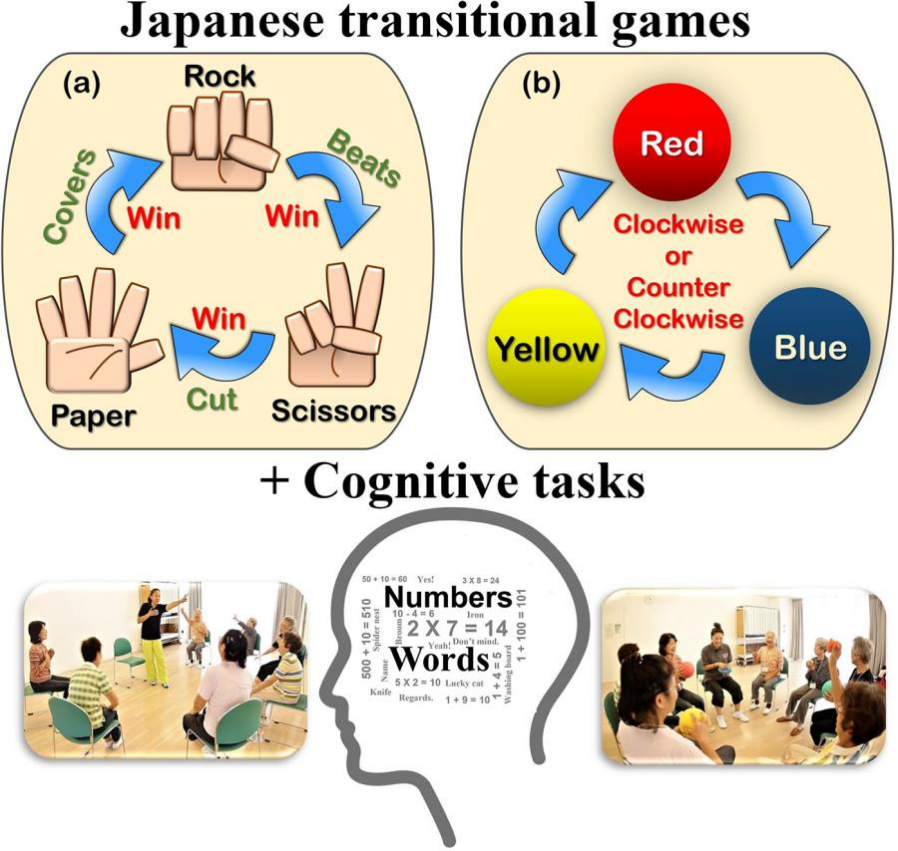
**Basic movements of “Synapsology” (games combined with number counting, calculation, memory, problem-solving, visual color recognition, enumerating words, etc.).** (**A**) First session: Rock, Paper, Scissors (RPS) game, (**B**) Second session: Pass the colored balls game. (See details in Supplementary Table 1).

SYNAP activates the brain’s cognitive functioning (calculation, memory/learning, attention/concentration, thinking/problem-solving, visual space recognition, and language, etc.) and its corresponding movements (Figure 1). However, the effects of SYNAP have not been verified, and this study is the first study to evaluate it quantitatively.

Considering the relationship of DT exercise and cognitive function, we hypothesized that a novel DT exercise method, Synapsology, would improve physical and cognitive functions of older adults. In the current pilot study, we comprehensively investigated both physical and cognitive functions in our subjects to assess the efficacy of SYNAP for older adults.

## RESULTS

### Participants and baseline characteristics

**Figure 2:**
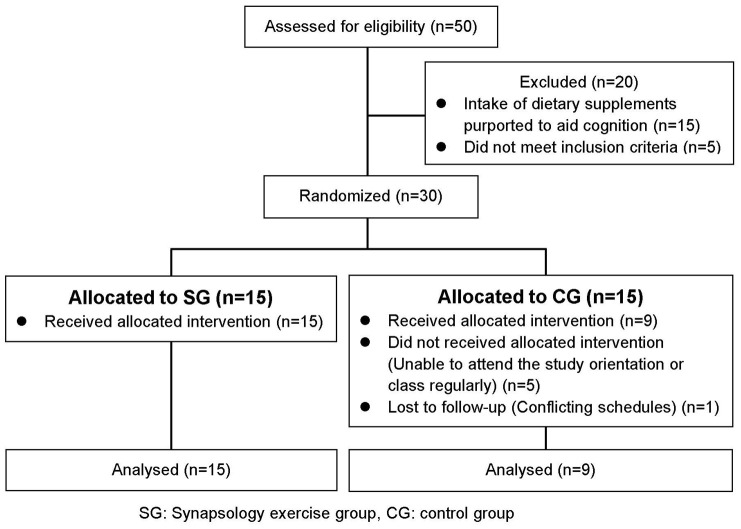
**Flow diagram of participants in the trial.**

A total of 50 older adults agreed to participate in our study (Figure 2). Among them, 20 applicants did not meet the inclusion criteria (intake of dietary supplements purported to aid cognition, having an acute phase of a disease). Thus, a total of healthy 30 older adults who lived in the local community participated in the study. During the training period, six participants (CG) dropped out during the physical function evaluation because of conflicting schedules or their participation in strength training twice a week. Twenty-four participants (SYNAP exercise group (SG): 15, Control group (CG): 9) completed the 8-week trial.

There was a significant difference in the number of education years (P=0.043) between the SG and CG (Table 1). Three participants in SG had a very high education background (19 years of education), which is the reason for the significant difference in the results. On the other hand, there were no significant differences in the baseline characteristics between groups. The mean ages of the participants were 69.7 years (65–76 years) for SG and 71.9 years (65–77) for CG. Their mean BMIs were 22.5 (17.8–26.8) for SG and 22.9 (21.1–25.5) for CG. The Japanese Ministry of Health, Labor, and Welfare (2013) lists the average BMI at 22.7 kg/m^2^ for adults between 60–69 years and 23.0 kg/m^2^ for those aged 70 years and above. Therefore, the participants’ average BMI was typical of the Japanese population for these ages.

**Table 1:** Participant characteristics.

**Items (unit)**	**SG (n=15)**				**CG (n=9)**				**P-value**
	**Mean**	**±**	**SD**	**Range**	**Mean**	**±**	**SD**	**Range**	
Age (years)	69.7	±	3.3	65-76	71.9	±	5.0	65-77	0.314
Women (%)	8 (53.3)				4 (44.4)	0.650^†^
Education (years)	14.6	±	3.0	9-19	11.9	±	1.6	9-14	0.043
Height (cm)	161.5	±	8.1	145.5-172.2	162.1	±	7.6	147.2-168.6	0.865
Body weight (kg)	58.8	±	9.6	47.2-71.0	60.6	±	8.9	49.2-72.1	0.674
Body mass index (kg/m^2^)	22.5	±	2.9	17.8-26.8	22.9	±	1.8	21.1-25.5	0.710
Systolic blood pressure (mmHg)	137.9	±	15.8	104-162	133.9	±	25.3	90-161	0.674
Diastolic blood pressure (mmHg)	77.4	±	8.5	63-93	78.1	±	9.2	69-92	0.854

**Medical history**			
Stroke	1 (6.7)	0 (0.0)	0.202^†^
Hypertension (%)	6 (40.0)	4 (44.4)	0.940^†^
Hyperlipidemia (%)	0 (0.0)	4 (44.4)	0.006^†^
Diabetes (%)	1 (6.7)	2 (22.2)	0.295^†^
Heart disease (%)	2 (13.3)	1 (11.1)	0.825^†^

SG: Synapsology exercise group, CG: control group, SD: standard deviation, P-value: Student's t-test, ^†^P-value: Chi-squared test

The mean blood pressures were similar with no significant difference between the two groups. The average blood pressure of older Japanese adults is 140/90 mm Hg, placing our participants’ mean blood pressures within the normal range. In addition, there was a difference between the groups in the history of hyperlipidemia alone, and four participants experienced this in CG. Six participants had a history of diabetes treatment (SG:1, CG:2) and heart disease (SG:2, CG:1).

### Performance of Synapsology (see details in Supplementary Table 1)

Synapsology is an 8-week DT intervention consisting of 60-minute sessions performed twice a week for a total of 16 intervention sessions. There were 240 participant- training hours available for SG (15 participants, 16 sessions). The members of SG attended 232 hours, which was a 96.7% attendance rate.

### Physical function

In Table 2, there were no significant differences in the timed-up-and-go (TUG, P=0.114), five times-sit-to-stand movement test (P=0.778), and 5-meter habitual walk (P=0.257) between groups (SG and CG). In the SG, the pre- and post- trial result showed a significant improvement after SYNAP intervention in TUG performance (P=0.017). There were no significant changes in the CG (P=0.779). In addition, the effect size (Cohen’s d) in the SG was “moderate” (d=0.51) in TUG and there were “no effect” in five times-sit-to-stand (d=0.10) and in the 5-meter habitual walk test (d=0.07). The effect size in CG was “small” in 5-meter habitual walk (d=0.44), “no effect” in TUG (d=0.03), and “small” in five times sit-to-stand (d=0.17).

**Table 2:** Physical and cognitive function by groups at baseline and follow-up.

**Item (unit)**	**SG (n=15)**						**CG (n=9)**						**^†^P-value(SG vs. CG)**
	**Pre-test**		**Post-test**		**Effect size (d)**	**P-value**	**Pre-test**		**Post-test**		**Effect size (d)**	**P-value**	
	**Median**	**Range**	**Median**	**Range**			**Median**	**Range**	**Median**	**Range**			
Physical function													
Timed up and go (sec.)	4.90	4.42-5.90	4.55	3.89-5.86	0.51	0.017*	4.89	4.23-7.97	5.00	4.22-6.90	0.03	0.779	0.114
Five-times-sit-to stand movement test (sec.)	5.89	4.59-7.96	5.47	4.55-8.67	0.10	0.950	5.73	4.95-6.64	5.54	4.79-7.88	0.17	0.678	0.778
5 m habitual walk test (sec.)	3.08	2.80-4.70	3.15	2.87-4.70	0.07	0.925	3.15	2.60-4.71	3.16	2.60-3.75	0.44	0.271	0.257
Cognitive function													
Trail making peg test (sec.)	68.51	51.75-99.75	54.38	46.02-95.50	0.54	0.004*	67.71	47.81-84.65	67.97	47.81-114.65	0.12	0.779	0.084
d-ROMs (U CARR)	394.00	307.00-476.00	389.00	285.00-486.00	0.28	0.039*	417.00	309.00-594.00	426.00	358.00-594.00	0.06	0.889	0.570
BDNF (ng/ml)	188.27	157.79-248.27	202.31	136.82-259.89	0.01	0.790	190.53	107.15-253.92	166.50	116.18-239.89	0.59	0.069	0.148

SG: Synapsology exercise group, CG: control group, Effect size (d): Cohen's d=|0.2≤d<0.5|=small, |0.5≤d<0.8|=moderate, |0.8≤d|=large. P-value: Wilcoxon signed-rank test (pre- vs. post- trial), ^†^P-value: Mann-Whitney u test (post Δ pre- trial). Asterisks show statistical significance at P<0.05.

### Cognitive function

### Trail-making peg test (TMPT, 25-hole peg test)

**Figure 3:**
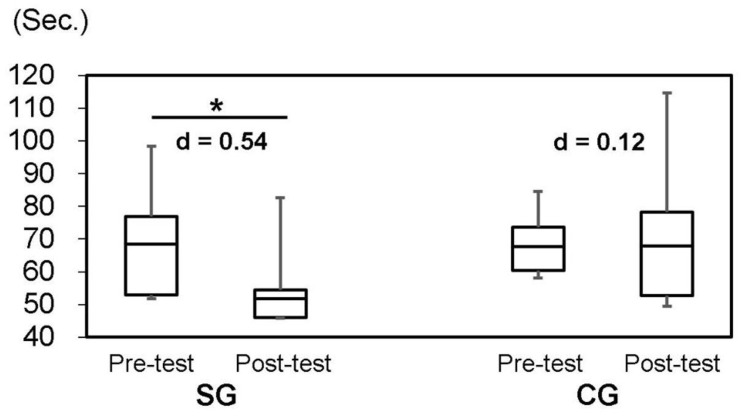
**Trail-making peg test (TMPT, 25-hole peg test) by groups at baseline and follow-up.** SG: Synapsology exercise group; CG: control group; Effect size (Cohen’s d): |0.2≤d<0.5|=small, |0.5≤d<0.8|=moderate, |0.8≤d|=large. Asterisk shows statistical significance at P<0.05.

In Table 2 and Figure 3, the results suggest a progressive improvement of TMPT (P=0.004) after SYNAP intervention. The differences on the TMPT between the SG and CG were not significant (P=0.084). However, the effect size of SG (d=0.54; moderate) was higher than that of CG (d=0.12; no effect).

### Oxidative stress markers (d-ROMs) and brain-derived neurotrophic factor (BDNF)

In Table 2 and Figure 3, the differences on d-ROMs and BDNF between groups were not significant (P=0.570 in d-ROMs and P=0.148 in BDNF). In SG, the result was significantly decreased in oxidative stress markers (d-ROMs) (P=0.039) after SYNAP intervention. In addition, the effect size (Cohen’s d) was “small” (d=0.28) in d-ROMs and “no effect” in BDNF (d=0.01). The effect size in CG was “moderate” in BDNF (d=0.59) and “no effect” in d-ROMs (d=0.06).

## DISCUSSION

In Japan, adults aged over 65 comprise 27.3% of the total population. Thus, the population structure has changed drastically to an elderly society. In 2000, the Nursing Care Insurance Act system was implemented to support various elderly problems in society as a whole. Half of the financial resources is secured by collecting insurance premiums from workers aged 40 and over, and the other half is covered by the government. In the system, the older adults with dementia need long-term care (so-called, Long-Term Care Insurance System), which imposes a heavy financial burden [19] on them.

The risk of cognitive impairment in older adults is a serious problem in Japanese society. Moreover, the older adults often experience medication problems. For example, they may experience medication side effects such as gastrointestinal problems, kidney damage or even kidney failure with the administration of medications such as painkillers and anti-inflammatory medications [3]. The most common illnesses among the elderly are heart and gastrointestinal problems, nerve damage, muscle pain and weakness, bone loss, and joint pain, etc. Consequently, the ideal solution for the QOL (Quality of Life) of older adults may be a combination of a non-pharmacological intervention such as a cognitive dual-task (DT) exercise and effective/natural supplements instead of medication.

In the study, thus, the cognitive and motor DT exercise program, Synapsology (SYNAP), was evaluated and its the beneficial effect for older adults was studied through physical and cognitive function measurements.

Previous studies reported that cognitive DT exercises indicated a high correlation between cognitive tasks and motor tasks [20, 21]. Our study using SYNAP was designed for older adults to participate in a DT exercise program with relatively complex task exercises based on Japanese transitional games. Thus, although the program consisted of 60-minute sessions over 8 weeks, the participants enjoyed the sessions and had a 96.7% participation rate. Participants expressed that they could continue this intervention because it was fun, like a game.

Although there was a significant difference in the education item (P=0.043) (Table 1) between the SG and CG, there was no significant difference in the timed-up-and-go (TUG, P=0.921), 25-hole peg test (TMPT, P=0.550), oxidative stress markers (d-ROMs, P=0.823), and brain-derived neurotrophic factor (BDNF, P=0.910) between groups, when considering education as a covariant. Therefore, education was not directly relevant for the items measured in this study. In other words, d-ROMs and BDNF levels in serum can be evaluated objectively as a cognitive function indicator. Indeed, most studies have typically used participants’ education (number of years/background) as a cognitive function covariant. Many cases showed that education was correlated with cognitive function [22, 23], but in some cases, they were not related to cognitive function [24, 25]. Therefore, it is still unclear whether education always affects cognitive changes or not.

The TUG provides more information regarding mobility and risk of falling because it measures cognitive capacity while performing a motor task [26]. In other words, TUG was strongly related to cognitive capacity and effectively performing the TUG depends on the person’s cognitive function. Although there was no significant difference in TUG, among the five times sit-to-stand movement test, and 5-meter habitual walk between groups (SG and CG), only the result of TUG in SG was significant (P=0.017). It suggests a progressive improvement of TUG in motor task exercise after SYNAP intervention. Therefore, SYNAP leads to an improvement in the participants’ TUG ability in connection with an improved cognitive function. However, SYNAP includes light daily movements and not high-intensity exercises. This 8-week intervention study did not increase the leg strength of the participants. Thus, the result of the five times sit-to-stand movement and the 5-meter habitual walk test in both groups were not improved by SYNAP intervention.

Kellor et al. originally introduced the peg test in 1971 as a measurement of dexterity [27]. It generally consists of a 9-hole peg board, which is considered vital for measuring dexterity. It is widely used in the assessment of cognitive function for neurological disorders together with a commercial version. More detailed information was gathered regarding the change in cognitive function from pre- to post- trial using a complicated 25-hole peg test called the “trail-making peg test” (TMPT). It was a fast and convenient tool for assessing cognitive function and is often used in Japan [28]. Okura and Yoon showed that the correlation coefficient of cognitive function was higher with this TMPT method (r=-0.682, P<0.001) than with the general trail-making peg test [29]. There was no significant difference between SG and CG in the 25-hole TMPT results. However, in SG, we observed a remarkable improvement (P=0.004). In addition, the effect size (Cohen’s d) was “moderate” (d=0.54) in SG, but there was “no effect” in CG (d=0.12). In other words, the median of SG after SYNAP intervention was increased but CG did not change (Figure 3). The results suggested that the participant’s brain was activated by SYNAP intervention, and movement speed in the task improved because of faster peg number recognition, since cognitive function had improved.

In a similar Japanese dual-task (DT) study [20], they conducted a calculation task, a visual search task and a verbal fluency task. The cognitive task was done simultaneously during the balance training in the DT group (n=21, 72.9 years) for twice a week within three months. Comparing the results of their DT, our TUG result was 1.2 times faster, and the TMPT result was 3.1 times faster. In our results, TUG and 25-TMPT showed a significant difference and the effect size also showed a “moderate” size difference (TUG: d=0.51, TMPT: d=0.54) after SYNAP intervention. However, the previous study showed no significant difference in those items and also, the effect size was “small”. This difference suggests that the results of physical and cognitive function may be influenced by the content of DT program rather than the DT intervention period. (i.e., it may be important that the elderly enjoy DT)

**Figure 4:**
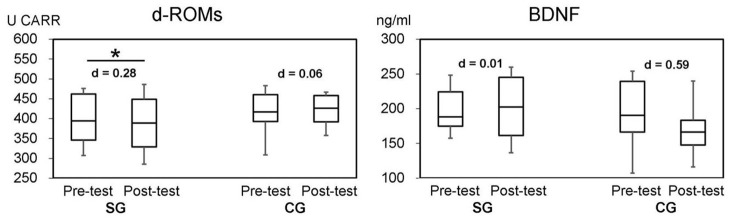
**Two bioassays (d-ROMs and BDNF) by groups at the baseline and follow-up.** SG: Synapsology exercise group; CG: control group. Effect size (Cohen’s d): |0.2≤d<0.5|=small, |0.5≤d<0.8|=moderate, |0.8≤d|=large. Asterisk shows statistical significance at P<0.05.

A simple method of detecting hydroperoxide levels in serum is to measure derivative-reactive oxygen metabolites (d-ROMs); this has been reported as a useful method for evaluating oxidative stress [30]. Oxidative stress and the level of serum oxidative stress markers are inversely associated with global cognition [31]. Thus, measuring d-ROMs is a simple and convenient assay method for assessing cognitive function changes. To our best knowledge, this is the first study to evaluate cognitive function changes using d-ROMs in DT intervention. As shown in Table 2, the levels of d-ROMs in both groups were not significant (P=0.570). However, the result of d-ROMs in SG was significant (P=0.039) after DT intervention (Figure 4). The effect size (Cohen’s d) was “small” (d=0.28) in SG, but there was “no effect” in CG (d=0.06). The levels of d-ROMs would indicate improved cognitive function, since d-ROMs are inversely associated with global cognition [31, 32]. Therefore, from the obtained result of TMPT and d-ROMs, we can conclude that SYNAP led to an improved cognitive function in SG compared to no SYNAP participants in the CG.

However, we did not see remarkable changes in BDNF. Interestingly, there was no change in the mean value of BDNF in SG, whereas it decreased in CG from 193.7 ng/ml in the pre- test to 168.6 ng/ml in the post- trial with “moderate” effect size (d=0.59). This decrease occurred in CG in only 8 weeks, but it may be impossible for cognitive function to decrease by such a level due to aging in such a short span of time. Without high-intensity exercise, there will be no change in BDNF level in blood serum. Thus, the BDNF levels in blood serum in this study were not affected or not measurable. Since BDNF responds to activities and stress that all individuals experience during their daily life, it might be difficult to distinguish changes in BDNF levels using the results from relatively short exercise/activity trials such as the DT trial.

Based on our results, this study suggests that SYNAP could be an effective DT program in adults aged over 65 years to improve and maintain their physical and cognitive function and to improve their quality of life. For this reason, SYNAP should be established as a social exercise in retirement homes and municipal sports facilities in Japan.

We could only include a small number of participants in this initial study, which was a limitation. We determined the number of participants we could accommodate in our study, in part, by considering our space requirements and availability in the university. Actually, we conducted SYNAP intervention in groups of 5 to 7 participants in a circle, and this required an area of 2.25 m^2^ or more per person. This study required not only activity space available for the exercise classroom, but also the number of skilled program instructors, nurses, and other assistants. This is the reason it was difficult to conduct a large-scale examination. Therefore, we cannot generalize the study findings as a system for the improvement of physical and cognitive functions for older adults.

Recently, a method was developed for quantitatively measuring amyloid beta (Aβ), a substance related to cognitive function changes, from a small amount of serum sample [33]. In our future studies, we will use this method to investigate the relationship between DT and cognitive function. Also, we plan to evaluate the effectiveness of Synapsology in the near future using fMRI or FDG-PET measurement.

## CONCLUSIONS

This research represents the first evaluation of a game-like DT exercise “Synapsology” (SYNAP). There was no significant difference between groups (SG and CG) in this study. However, by participating in SYNAP, older adult participants exhibited positive changes in the TUG, TMPT, and d-ROMs but not in BDNF levels. The participants’ motor ability and cognitive function were better than before they followed SYNAP.

This study suggests that participation in SYNAP for over 8 weeks may slow or prevent cognitive deterioration in older adults as compared to no intervention. Therefore, SYNAP would be a useful addition to the social and recreational activities of older adults, especially in a “super-aging” society like Japan.

## MATERIALS AND METHODS

### Participants and baseline characteristics

The older adults, who participated in this study, were recruited through an advertisement in the local newspaper (JOYO Living). The inclusion criteria for participants were adults aged over 65 years who did not regularly engage in organized physical activities, sports, or cognitive training. The power analysis with settings at α=0.05, power (1–β)=0.80 and effect size=0.64 [34] showed that a group of 30 participants was the required sample size for SG. However, since this was a pilot study, only half of the 30 people were sampled. Initially, patients with dementia were excluded. The exclusion criteria were as follows: (1) those using a pacemaker; (2) those undergoing an acute phase of a disease; (3) those with severe diabetes; (4) those participating in strength training two or more times per week; or (5) those unable to attend the study briefing or continuously attend the training sessions. In Figure 2, the CONSORT flow chart provides more information on participant flow and distribution. We enrolled 30 healthy participants in the trial and put them into two groups randomly using the blocked randomization method [35]. The information collected from the participants included baseline characteristics such as age, height, weight, body mass index (BMI), and blood pressure (Table 1).

The Ethics Committee of the University of Tsukuba approved this study (No., Tai 26-95). All participants provided written, informed consent. The authors confirm that the trials for this intervention were registered with the University Hospital Medical Information Network (UMIN) Clinical Trial Registry (UMIN000034350). The experimental procedures were fully explained to each participant before they gave their written informed consent. All data are available from the University of Tsukuba Ethics Committee for researchers who met the criteria for access to confidential data.

### Synapsology program (see details in Supplementary Table 1)

Synapsology (SYNAP), an advanced DT program for stimulating brain activity, was developed in 2011 [15]. It has been applied, in particular, to older adults, including those living in group homes and participating in cognitive support programs.

The SYNAP program uses physical and mental game-playing in a multidisciplinary approach to stimulate brain activity and cognitive performance, as well as improve physical function. In Japan, “Janken,” or the rock-paper-scissors (RPS) game as it is called in other parts of the world, was a very popular and familiar traditional game among children and even adults. It was helpful and desirable for the DT program to be simple and enjoyable like a game (Figure 1). The SYNAP program has the participant play physical games mildly using colored balls or make the RPS symbols with the left and right hands while performing a series of cognitive tasks; they may need to call out related or memorized words or count and add numbers while tossing a ball or making the RPS symbols. Stretching, lower body exercises, and walking were included in our SYNAP program.

The program consisted of 60-minute sessions twice a week as an intervention for 8 weeks. The sessions were conducted with in groups of five to seven participants with one instructor, to maintain their concentration. The SG begins with a 10-minute warm-up of breathing and flexibility exercises for the upper and lower body, followed by a 45-minute SYNAP program. Then, the program ends with a 5-minute cooling down with breathing and stretching exercises. The first main session was an RPS game, and the second main session was a “pass the colored balls” game with exercises, calculations, words, memorization, and other tasks. The first and second sessions were conducted on the same day. It was structured so that the difficulty level of the task became higher each week or two. The first main session consisted of an RPS game (1^st^–2^nd^ week), RPS calculation exercise (3^rd^ week), confrontation exercise (4^th^–5^th^ week), RPS variant-calculation (6^th^ week), memory RPS (7^th^–8^th^ week), and the second main session consisted of “pass the colored balls” game (self-introduction) (1^st^–2^nd^ week), four movements (housework) (3^rd^ week), four actions with a partner (4^th^–5^th^ week), partner hand touch (6^th^ week), and mimicry with a partner (7^th^–8^th^ week). In total, our participants attended 16 sessions. The CG did not receive the intervention at all, but they performed normal and regular activities of daily living during the study period. The purpose of the CG was to compare changes in the physical and cognitive functions of elderly participants who did not perform the SYNAP program with those who did.

### Physical function evaluation

### Timed-up-and-go-test (TUG)

TUG is a commonly used performance test to evaluate functional mobility or dynamic balance. According to the modified method [36], the participants were instructed to rise from their chair, walk 3 m to a marker, go around it, and sit down on a chair as fast as possible. The time taken to complete the task was recorded; the faster time of the two trials was used for the analysis.

### Five times sit-to-stand movement test

The participants were asked to sit on their chair with arms folded over their chests and then to stand up and sit down 5 times as fast as possible.

### 5-m habitual walk test

Participants walked at their typical speed on an 11-m straight course. To eliminate acceleration phases, we calculated the walking time between the 3-m and 8-m marks of the course. The time taken to complete the task was recorded, with the faster time among the two trials was used for the analysis.

### Cognitive function evaluation

### Trail-making peg test (TMPT, 25-hole peg test)

**Figure 5:**
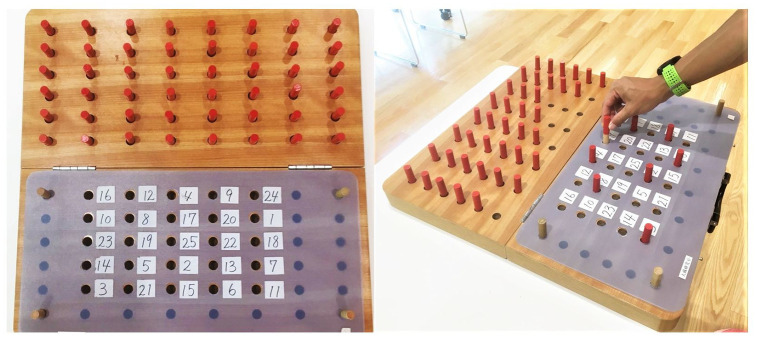
**Trail-making peg test (TMPT, 25-hole peg test) kit.**

In the TMPT, the participants were asked to place 25 pegs in 25 randomly numbered holes on a board in numerical order from 1 to 25 and subsequently remove them as quickly as possible (Figure 5). This requires a visual-scanning task to locate the numbered holes and move the 25 pegs. We used the TTKK 1306 peg board (Takei Scientific Instruments Co. Ltd., Niigata, Japan), and recorded the time required for participants to finish moving 25 pegs.

### BDNF and d-ROM measurements

We collected blood from the participants of both groups, a BDNF assay as a marker for cognitive function, and a d-ROMs assay as an oxidative reaction indicator, in the morning after fasting overnight. We collected the blood and ran these tests about 1 week prior to the intervention (pre- trial, measurement point 1) and about 8 weeks after starting the intervention (post- trial, measurement point 2).

### Measurement of brain-derived neurotrophic factor (BDNF)

After collecting 4 mL of blood from each participant by venipuncture, the blood was allowed to coagulate at room temperature for 30 minutes and was subsequently centrifuged for 15 minutes at 3000 rpm to collect the serum. We performed an enzyme-linked immunosorbent assay (ELISA) to quantitatively determine the BDNF concentrations (DuoSet ELISA Development Kit from RD Systems, AVISCERA BIOSCIENCE, INC.). No significant cross reactivity or interference was observed in this assay. Serum samples were diluted 1:50. The samples of BDNF concentrations were then determined by non-linear regression from the standard curves. We prepared “low” and “high” concentration quality control pools by adding 10 ng and 100 ng to 5 ml portions of human serum (Innovative Research, Novi, MI, USA), giving us nominal concentrations of 2 and 20 ng/ml, respectively. The repeatability of the BDNF ELISA, as measured by intra-assay precision, was 3.8%, and the reproducibility, as measured by inter-assay precision, was 7.6%.

### Measurement of serum oxidative stress markers (d-ROMs)

The serum concentrations of d-ROMs were used to assess oxidative stress markers. Twenty microliters of serum were added to a pH 4.8 buffer in a cuvette and mixed by inversion to separate Fe2β and Fe3β from the blood proteins and allow the Fe2β and Fe3β radicals to catalyze the degradation of hydroperoxides in the blood into alkoxyl and peroxy radicals, to measure the d-ROMs level. Next, when 20 mL of a coloring chromogen (N, N-diethyl-paraphenylenediamine) was added to the solution, the free radicals oxidized the chromogen substrate to yield red-colored radical cations. The solution was mixed again by inversion, and the cuvette was placed in a photometer for optical measurement at 505 nm.

### Statistical analysis

The G*Power 3.1.3 was used to perform the power analysis for the supposed sample size. All data were checked for normality of distribution with the Shapiro-Wilk test.

We used baseline characteristics as the mean and compared it to standard deviation or the frequency count between the groups using Student’s t-test and χ^2^ test.

Comparisons of the baseline values with those 8 weeks after each exercise training session were performed using the nonparametric Wilcoxon signed-rank test for each group. We also performed comparisons between both the groups using the nonparametric Mann-Whitney U test. A Δ value was obtained from the Mann-Whitney U test by subtracting post- trial from pre- trial and was used to estimate whether the changes in the parameters were clinically meaningful and to examine the effect size. The effect sizes (Cohen’s d) of the pre- and post- trial data were determined using the average changes and excluding the pre- test standard deviation. A value of 0.2–0.5 was considered “small;” 0.5–0.8, “moderate;” and above 0.8, “large”. In addition, 95% confidence intervals were used for all resulting measures of the pre- and post- trial. Data were analyzed using the Windows statistical package for IBM SPSS Statistics 22 software (IBM, Armonk, NY, USA) with the level of statistical significance set at P<0.05.

## Supplementary Material

Supplementary Table 1
