# Supplementary material for: Evaluation of beneficial effect of a dual-task exercise based on Japanese transitional games in older adults: a pilot study
Source: Aging (Albany NY). 2020 Oct 11;12(19):18957–69. doi: 10.18632/aging.103908 (PMC7732331; doi:10.18632/aging.103908)
Supplement: Supplementary Table 1 [file aging-12-103908-s001..pdf]

Supplementary Table 1. Synapsology program.

<table>
<tr><th colspan="2">Warming up (10 min.) + Synapsology exercise (3 sessions × 15 min. = 45 min.) + Cooling down (5 minutes) = 60 min.</th></tr>
<tr><th>Week(s)</th><th>1st SESSION</th></tr>
<tr><td rowspan="2">1-2</td><td>Rock, Paper, Scissors game (RPS) where rock beats scissors, scissors cut paper and paper covers rock</td></tr>
<tr><td>
1. During the first part of the game, participants try to win against the instructor showing their hand symbol and saying their choices out loud. (5 sets)

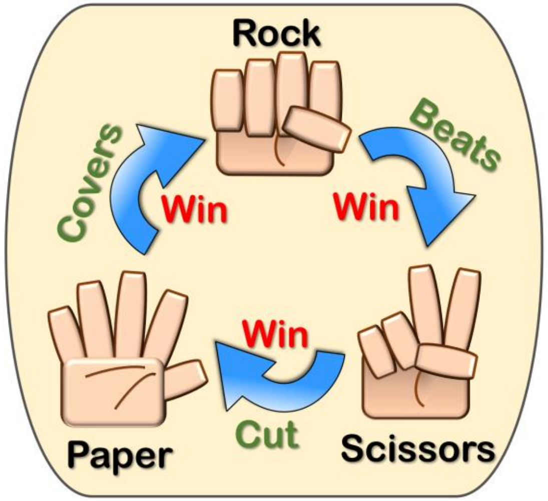

2. Next, participants try to lose against the instructor using the same process. (3 sets)
3. In the next stage, participants use both hands to display the 2 choices the instructor did NOT make (e.g., Instructor shows paper, participants show scissors on one hand and rock on the other). (5 sets)
4. In the last stage, participants gesture the winning choice on the right hand and the losing choice on the left (e.g., instructor--scissors; participant--rock on right and paper on left). (5 sets)
</td></tr>
</table>

RPS Calculation Exercise

3

Participants and instructor play RPS while saying the name of their hand symbol. However, in this game, each hand symbol corresponds to a particular value.

1. Participants must add their displayed RPS symbol value with the instructor’s symbol value and say the answer out loud.（3 sets）

	Rock	Scissors	Paper
value	1	2	5

2. Participants repeat exercise #1, using different values for each symbol.（5 sets）

	Rock	Scissors	Paper
value	10	20	50

3. Participants repeat exercise #1 with another new set of symbol values. （4 sets）

	Rock	Scissors	Paper
value	1	20	500

4-5	**Confrontation Exercise**<br>1. First, participants say out loud the name of each finger on their hands starting from the thumb to the index finger, middle finger, ring finger and little finger. The process and naming is reversed on the opposite hand. After that, participants count 1 to 10 out loud using their fingers. (3 sets)<br>2. Next, participants count from 1 to 10 starting with the little finger on the right hand but skipping the thumb on the right so number 5 is the thumb on the left hand continuing on up to the little finger which is counted twice as numbers 9 and 10. (3 sets)<br>3. Finally, participants should repeat exercise #1 without looking at their fingers. (3 sets)
6	**RPS variant--calculation**<br>1. Participants make the paper symbol on their right hands holding it out in front of them while simultaneously making the rock symbol with their left hands and placing it on their chests. When the instructor says, "Yes!" participants exchange the symbols between their hands and hold them in the appropriate position (i.e. forward hand with paper symbol and rock symbol on the chest. (3 sets)<br>2. Next, the instructor says a number (1 through 5), and the participants hold out the correct number of fingers on the chosen hand while the other is placed on their chests in the rock symbol. (4 sets)<br>3. The instructor again calls out a number. The participants now must hold up the number of fingers on their outstretched hand to obtain a sum of 5 when added to the number called out by the instructor while simultaneously placing the other hand in a rock symbol on their chests. For example, the instructor says 1, and the participants show 4 fingers on the outstretched hand (1 + 4 = 5). (4 sets)<br>4. Finally, the instructor says a number, and participants hold up fingers on both hands that, when summed, will equal the desired number. For example, for number 4, one hand shows 1 finger, the other shows 3. One hand still remains outstretched and the other is on the chest. (3 sets)

	**Memory RPS**
7-8	1. The instructor shows a RPS symbol on her hand after chanting "rock, paper, scissors." participants must make the last RPS symbol that the instructor showed and say it out loud. (4 sets)<br>2. Next, participants must remember the previous symbol shown by the instructor, but this time they must form the symbol that would WIN against the instructor's previous symbol and say it out loud. (4 sets)<br>3. Following the same process as exercise #2, participants should show the symbol that would LOSE against the instructor's previous choice and say it out loud. (4 sets)<br>4. Again following the same process as exercise #2, this time the participants display the symbol on their right hands that would WIN against the instructor's choice and show the symbol on their left hands that would LOSE against the instructor's choice. (4 sets)

<table>
<tr><th>Week(s)</th><th>2nd SESSION</th></tr>
<tr><td rowspan="3">1-2</td><td>Pass the colored balls game (Self-introduction)</td></tr>
<tr><td>1. Participants sit on chairs forming a circle with 3 people holding either a red, yellow or blue ball. When the instructor says, “Yes,” each ball is passed clockwise. This continues in the same direction until the instructor says "Stop," at which point each ball is passed counterclockwise. (2 sets)
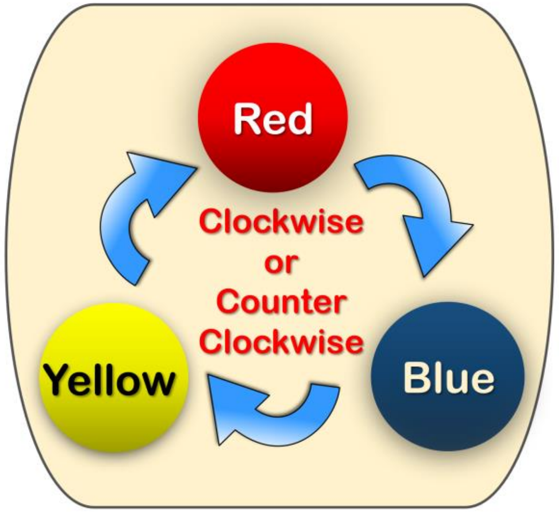
</td></tr>
<tr><td>2. During the next phase, the person holding the red ball when the instructor says "Stop" must say her family name before passing it in the opposite direction. (4 sets)
3. Next, as the balls are passed from person to person, whoever is holding the yellow ball when the instructor says, "Stop," must say her given name before passing it in the opposite direction. (4 sets)
4. Again the balls are passed, but this time, the participant left holding the blue ball must say her nickname before passing it in the opposite direction. (4 sets)</td></tr>
</table>

<table>
<tr><td rowspan="2">3</td><td>Four movements (housework)</td></tr>
<tr><td>The participants are given four actions to remember corresponding to the following numbers and household tools:
<table>
<tr><td>No. 1</td><td>Broom</td><td>Place both hands on your head</td></tr>
<tr><td>No. 2</td><td>Iron</td><td>Cross arms and place both hands on your shoulders</td></tr>
<tr><td>No. 3</td><td>Knife</td><td>Place both hands on your waist</td></tr>
<tr><td>No. 4</td><td>Wash board</td><td>Cross arms and place both hands on your knees</td></tr>
</table>
1. The instructor says a number and the participants perform the corresponding action from the table. (3 sets)
2. In the next phase, the instructor names one of the tools, and the participants must perform the corresponding action from the table. (3 sets)
3. Next, the instructor pretends to use one of the tools, and the participants say the tool’s name out loud and exhibit the corresponding action from the table. (3 sets)
4. Finally, the instructor either says a number or pretends to use one of the tools. The participants must say the tool’s name out loud and exhibit the action corresponding to it in the table. (3 sets)</td></tr>
<tr><td rowspan="2">4-5</td><td>Four actions with a partner</td></tr>
<tr><td>The participants are given four actions to remember corresponding to the following numbers and phrases:
<table>
<tr><td>No.1</td><td>“Yeah!”</td><td>Touch both hands to both of partner’s hands.</td></tr>
<tr><td>No.2</td><td>“Don’t mind.”</td><td>Use right hand to shake partner’s right hand.</td></tr>
<tr><td>No.3</td><td>“Regards.”</td><td>Use left hand to shake partner’s left hand.</td></tr>
<tr><td>No.4</td><td>Say name of partner</td><td>Touch partner’s outer right thigh with both hands.</td></tr>
</table></td></tr>
</table>

	1. The instructor says a number and the participants perform the corresponding action.（4 sets）<br>2. Next, the instructor calls out 2 numbers and the partners successively perform the actions corresponding to each number.（4 sets）<br>3. Finally, when the instructor says a number, the partners must say the phrase associated with it while performing the appropriate action（6 sets）
6	**Partner hand touch**
	1. The participants are divided into two groups. In the first exercise, all participants in group 1 extend their hands showing their palms to everyone while moving about the room. Members of group 2 then touch the palms of the group 1 members while saying "Yes!" out loud. (4 sets)<br>2. Next, each participant shows either a rock or paper hand symbol and participants from group 2 must link their hand symbols to the same symbol of a member in group 1. (6 sets)<br>3. Again, participants show either a rock or paper symbol, but this time, group 2 members should link their symbols to win against group 1 members. (6 sets)<br>4. The process in exercise # 2 is repeated (matching the same symbol), but this time when their hands are linked, they will say "Spider nest" for linking rocks and "Lucky cat" for linking paper (6 sets).
7-8	**Mimicry with a partner**
	1. Participants are paired off and choose to be either partner #1 or #2. Partner #1 should hold a body part of partner #2 using both hands while saying "Yes." Partner #2 will imitate the same action on #1. (6 sets)<br>2.Next participant #1 makes a random pose while saying "Yes!" with each pose, partner #2 must remember and imitate the last pose of partner #1. (4 sets)<br>3. Finally, partner #2 must imitate the last 2 poses of his partner. (4 sets)

<table>
<tr><th>Week(s)</th><th>3rd SESSION</th></tr>
<tr><td rowspan="2">1-2</td><td>Partner ball pass</td></tr>
<tr><td>1. Partners are seated facing each other while holding up a ball in their right hands. They count to10 and place the ball on their own laps. (4 sets)
2. The partners start in the same manner as in exercise #1, but this time, when they reach numbers 3 and 7 in their counting, they exchange balls. (4 sets)
3. Proceed as in exercise #2, but in addition to exchanging balls at 3 and 7 in their counting, the participants touch their balls in the middle when they reach number 5. (4 sets)
4. Repeat exercise #3 with the addition of saying "Yes." when they touch their balls together. (6 sets)</td></tr>
<tr><td rowspan="2">3</td><td>Partner ball pass and counting numbers</td></tr>
<tr><td>1. The participants are given four actions to remember corresponding the following number:
<table>
<tr><td>No.1</td><td>Place both hands on the head.</td></tr>
<tr><td>No.2</td><td>Cross both hands on the shoulder.</td></tr>
<tr><td>No.3</td><td>Place both hands on the waist.</td></tr>
<tr><td>No.4</td><td>Cross both hands on the knees.</td></tr>
</table>
The instructor will give out a number and the participants will show the corresponding action associated with it. (4 sets)
2. The same process is done on the exercise #1, but this time the instructor will say out names of tools instead of numbers.
<table>
<tr><td>No.1</td><td>No.2</td><td>No.3</td><td>No.4</td></tr>
<tr><td>Broom</td><td>Iron</td><td>Knife</td><td>Washing board</td></tr>
</table>
</td></tr>
</table>

	When the instructor says the name of the tool out loud, the participants are expected to exhibit a corresponding action. (4 sets)<br>3. The instructor will show an action representing the tools in the exercise #2 and the participants are expected to guess the tool out loud and exhibit the action. (4 sets)<br>4. The instructor will either give a number or motions an action corresponding a tool, the participants need to guess the tool out loud and exhibit the action corresponding it. (6 sets)
	**Counting and ball passing**
4-5	1. Participants sit in 2 rows facing each other, each holding a ball in the air as they count from 1 to 10. Upon reaching number 10 they place the ball into the lap of the person on their right. (2 sets)<br>2. Next, as each person holds up the ball and counts, they must pass the ball to the person on the left when they reach number 3. (2 sets)<br>3. This time as they count and hold up the ball, they pass the ball to the left when they reach number 7. (2 sets)<br>4. Finally, as the participants count, they pass the ball across to the person facing them when they reach number 5. (4 sets)
	**Four actions with a partner**
6	The participants are given four actions to remember corresponding the following number:

No.1	“Yeah!”	Touch both hands to both of partner’s hands.
No.2	“Don’t mind.”	Use right hand to shake partner’s right hand.
No.3	“Regards.”	Use left hand to shake partner’s left hand.
No.4	Say name of partner	Touch partner’s outer right thigh with both hands.

	1. The instructor says a number and the participants perform the corresponding action.（4 sets）<br>2. Next, the instructor calls out 2 numbers and the partners successively perform the actions corresponding to each number.（4 sets）<br>3. Finally, when the instructor says a number, the partners must say the phrase associated with it while performing the appropriate action（6 sets）
7-8	**Hands and feet rhythm**
	1. While sitting down, participants repeatedly say "TONTON" while moving a heel up and down and tapping their thighs alternately with their right and left hands in rhythm to the "TONTON" beat. (4 set)<br>2. This time, they move a heel up and down and say "TENTEN" while tapping one thigh with both hands simultaneously. (4 set)<br>3. During either exercise above, when the instructor claps her hands once, the participants should perform the “TONTON” rhythm maneuver. If the instructor claps her hands twice, the participants should speed up the beat. (6 set)
